# Racial–Ethnic Disparities of Obesity Require Community Context-Specific Biomedical Research for Native Hawaiians and Other Pacific Islanders

**DOI:** 10.3390/nu16244268

**Published:** 2024-12-11

**Authors:** Riley K. Wells, Amada Torres, Marjorie K. Mau, Alika K. Maunakea

**Affiliations:** 1 Department of Molecular Biosciences and Bioengineering, College of Tropical Agriculture and Human Resources, University of Hawai‘i at Mānoa, Honolulu, HI 96822, USA; 2 Department of Anatomy, Biochemistry, and Physiology, John A. Burns School of Medicine, University of Hawai‘i at Mānoa, Honolulu, HI 96822, USA; torres91@hawaii.edu; 3 Department of Native Hawaiian Health, John A. Burns School of Medicine, University of Hawai‘i at Mānoa, Honolulu, HI 96813, USA

**Keywords:** gut bacteria, obesity, 16S metagenomics, dysbiosis, metabolic diseases, type 2 diabetes, health disparity, NHPI, indigenous

## Abstract

Compared to the general population of Hawai‘i, Native Hawaiians and Other Pacific Islanders (NHPI) shoulder a disproportionately high risk for obesity-related cardiometabolic disorders, such as type 2 diabetes and cardiovascular disease. The gut microbiome is an area of rapid research interest for its role in regulating adjacent metabolic pathways, offering novel opportunities to better understand the etiology of these health disparities. Obesity and the gut microbiome are influenced by regional, racial–ethnic, and community-specific factors, limiting the generalizability of current literature for understudied populations. Additionally, anthropometric and directly measured obesity indices are variably predictive of adiposity and metabolic health risk in this diverse population. Thus, further NHPI-inclusive research is required to adequately characterize community-specific factors in the context of obesity-related disease etiology. Culturally responsible research ethics and scientific communication are crucial to conducting such research, especially among indigenous and understudied populations. In this review, we explore these limitations in current literature, emphasizing the urgent need for NHPI-inclusive research to assess community-specific factors accurately. Such accuracy in Indigenous health research may ensure that findings relevant to individual or public health recommendations and/or policies are meaningful to the communities such research aims to serve.

## 1. Introduction

In the United States, Native Hawaiians and other Pacific Islanders (NHPI) are more likely to be classified as “obese” compared to other racial–ethnic groups, with an estimated 45.6% of NHPIs qualifying for obesity by conventional standards in 2022 [1]. Obesity is a multifaceted risk factor for several serious health complications, including cardiovascular disease (CVD), type 2 diabetes mellitus (T2DM), and cancer [2,3,4], conditions which are especially prevalent in NHPI communities and are among the leading causes of death in Hawai‘i [5]. This disparity is exacerbated by the underrepresentation of NHPIs in biomedical research, a gap contributing to a lack of targeted health interventions for this population [6,7].

The link between excess fat accumulation and cardiovascular health is mediated by chronic low-grade inflammation and its associated disruption of metabolic processes (termed “meta-inflammation”) [8]. Although adipocytes play an intricate role in normal homeostatic functioning [9], sustained excess adiposity may lead to cytokine and adipokine dysfunction, increased recruitment and infiltration of proinflammatory immune cells, and further proinflammatory polarization of tissue-resident immune cells [10]. The persistence of such inflammation interferes with critical pathways involved in glucose and lipid homeostasis [11]. Unresolved hyperglycemia and dyslipidemia that accompany chronic meta-inflammation can lead to several serious health complications, including CVD, T2DM, and cancer [3,12,13,14].

In the pursuit of identifying more targeted strategies to address these health complications, the gut microbiome has become of increasing translational research interest for its capacity to modulate inflammation and metabolism [15]. Several modes of fecal microbiota transplantation (FMT) are safe, practical strategies for reversing meta-inflammation, restoring glucose tolerance and lipid homeostasis, and even reducing visceral adiposity, demonstrating the functional relevance of a “healthy” or “unhealthy” (“dysbiotic”) gut microbiome [16]. Dietary and behavioral factors, including prebiotic and probiotic supplements, are also associated with distinct changes among gut bacterial populations. This presents a non-invasive intervention opportunity leveraging the bidirectional nature of host–microbe interactions [16,17].

Although obesity-related gut microbiome research may offer a promising avenue for mitigating risk for metabolic dysfunction, its generalizability in understudied populations is limited by two major factors: (1) the generalized standards for designating obesity status, largely based on BMI, which may mischaracterize obesity-related health risks in underrepresented populations [18], and (2) the regional and racial–ethnic differences that may impact gut microbiome composition. In this review, we explore these limitations in the context of health disparities research, highlighting the need to expand NHPI-inclusive studies to consider both community- and individual-specific factors. The mechanistic underpinnings of gut bacterial interactions with obesity-related disease etiology are complex and incompletely understood in NHPI populations.

## 2. The Definition of Obesity Is Not Universal

### 2.1. International BMI Standards for Obesity Diagnosis

According to the World Health Organization (WHO), obesity is abnormal or excessive fat accumulation that presents a risk to health [19]. More specifically, excess adiposity may be causally linked to CVD, heart disease, stroke, T2DM, and osteoarthritis [20]. The standard metric for designating obesity status in adults is body mass index (BMI), calculated as weight (kg) divided by square height (m^2^), using the following categorical cutoffs recommended by the WHO: underweight (BMI < 18.5 kg/m^2^), normal weight (18.5 kg/m^2^ ≤ BMI < 25 kg/m^2^), overweight (25.0 kg/m^2^ ≤ BMI < 30 kg/m^2^), Obesity Class I (30 kg/m^2^ ≤ BMI < 35 kg/m^2^), Obesity Class II (30 kg/m^2^ ≤ BMI < 35 kg/m^2^), and Obesity Class III (BMI > 40 kg/m^2^) [21]. According to these standards, an individual with a BMI over 25 kg/m^2^ or 30 kg/m^2^ would face a heightened risk for chronic metabolic and cardiovascular disorders compared to an individual with a BMI between 18.5 and 25 kg/m^2^, which is expected to rise as BMI increases [21].

Although BMI-related health risk assessments are commonplace in clinical and research settings [18], the utility of BMI as a universal tool for assessing health risk is limited. The original formulation for BMI was determined using observational studies in a sample of healthy men of European ancestry [18,22]. Therefore, a universal definition of obesity based solely on BMI may not be an equally effective indicator of metabolic disease risk related to adiposity.

**Figure 1:**
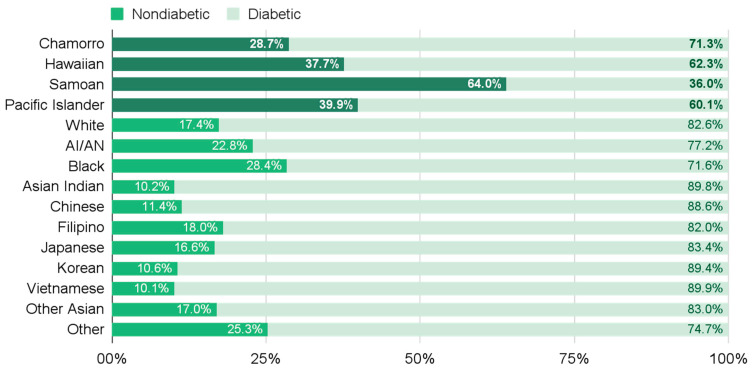
Prevalence of nondiabetic status in individuals with BMI ≥ 30 kg/m^2^ in Hawai‘i by racial–ethnic group (2022) [1]. AI/AN = American Indian and Alaska Natives.

This discrepancy can be observed among diverse populations in the State of Hawai‘i. In 2022, NHPIs had the highest rate of obesity compared to all other census race groups. However, among Hawai‘i state residents who were classified as “obese” (BMI ≥ 30 kg/m^2^), NHPIs (including Chamorro, Hawaiian, Other Pacific Islander, and especially Sāmoan populations) had the highest proportion of nondiabetic individuals (Figure 1) [1]. This demonstrates that the conventional definition of obesity is limited as a proxy measure for metabolic risk across racial–ethnic groups in this community, and it is particularly limited in NHPI populations in Hawai‘i. More importantly, this discrepancy suggests that metabolic health risk may be commonly mischaracterized in NHPI and other minority populations.

In 2004, the WHO Expert Committee acknowledged racial–ethnic differences in obesity and BMI and identified “public health action points…along the continuum of BMI” (23.0, 27.5, 32.5, and 37.5 kg/m^2^) at which a community may consider reevaluating cardiometabolic health risk while retaining traditional BMI cutoffs as the international diagnostic standard for overweight and obesity [23]. As such, independent institutions have recommended ethnic-specific BMI cutoffs to mitigate intergroup discrepancies (Table 1) [22,23,24,25,26,27,28,29]. According to the Korean Society for the Study of Obesity, such modifications have even been integrated into clinical practice guidelines for some Asian countries [28].

In 2000, the International Diabetes Institute (IDI) proposed modified BMI cutoffs to designate obesity status among Asian populations in the Asia-Pacific region. Under these standards, overweight and obese are designated at BMI ≥ 23 kg/m^2^ and ≥25 kg/m^2^, respectively, with increasing risk for cardiometabolic comorbidities with increasing BMI. The same publication acknowledged that this definition of obesity is not necessarily applicable to other communities in the Asia-Pacific region [26]. Pacific-Islander (PI)-specific BMI cutoffs proposed by the IDI for overweight and obesity were 26 kg/m^2^ and 32 kg/m^2^, respectively. These thresholds were developed in a 1999 study that measured body fat mass at fixed BMI values in European individuals. Those body fat measurements were then used as fixed values to back-calculate equivalent BMI cutoffs for a Polynesian (Māori and Sāmoan) cohort [30].

Although it is unclear whether these Polynesian-specific standards have been implemented in clinical settings, a 2010 study revisited these standards and found that the increased threshold for obesity at 32 kg/m^2^ did not significantly improve the utility of BMI as a predictor for insulin resistance or metabolic syndrome (MetS) in Māori populations [31]. The correlation between body fat and cardiometabolic risk is inequivalent between communities [32], an assumption that served as a basis for proposing a 32 kg/m^2^ obesity threshold for Polynesian populations. Consideration of such discrepancies is necessary to mitigate inaccurate characterizations of health in community education and data dissemination of research findings, especially for otherwise understudied populations.

### 2.2. Body Composition as a Metabolic Health Risk Factor

Differences in the relationship between body fat and metabolic health are sometimes attributed to individual-specific patterns in body composition, which can be measured directly [33]. Bioelectrical impedance analysis (BIA) can be used to estimate total body fat, fat mass, lean mass, and bone mineral density, among other parameters [34]. Imaging-based techniques, such as dual-energy X-ray absorptiometry (DXA), can estimate total and regional body fat distribution, and magnetic resonance imaging (MRI) is effectively able to differentiate between subcutaneous adipose tissue and visceral adipose tissue [35]. Applying such techniques has led to the establishment of a causal link between abdominal fat and metabolic dysfunction in some populations [35,36].

However, even direct measures of adiposity lack universality as a tool for health risk assessment due to racial–ethnic differences in obesity-related disease etiology [36,37]. In a multiethnic cohort (MEC) study, the association between MetS risk and percent body fat was weaker, and that between MetS risk and visceral adiposity tissue (VAT) analysis by MRI was stronger in Native Hawaiian individuals than in White individuals. More specifically, different regions of the visceral adipose tissue compartment were differentially associated with MetS risk [38]. These trends have sparked a controversial debate about whether specific populations preferentially deposit abdominal fat [23].

Alternative anthropometric indices have been designed to reflect adiposity more accurately. Such indices include waist circumference (measured in cm), waist-to-hip ratio (waist circumference divided by hip circumference), waist-to-height ratio (waist circumference divided by height, measured in the same units), waist-to-height^0.5^ ratio (WHT.5R; (waist circumference in cm)/(height in cm)^0.5^) [39], a body shape index (1000 × (waist circumference in m) × (weight in kg)^−2/3^ × (height in m)^5/6^) [40], body adiposity index ((hip circumference in cm)/((height in m)^1.5^) − 18) [41], and body roundness index (364.2 − 365.5 × (1 − ((0.5 × (waist circumference in m)/π)^2^/(0.5 × (height in m))^2^))^0.5^) [40]. These anthropometric indices vary in their effectiveness as indicators for adiposity. At a fixed BMI, Māori and other Pacific Islanders (PI) tend to have less total percentage of body fat but significantly more accumulated fat in the visceral adipose tissue compartment [30,42]. Specifically, a New Zealand study found that BMI was differentially associated with body fat mass (measured by dual-energy X-ray absorptiometry) by gender and race/ethnicity [30]. The association was highest for Māori men (R^2^ = 0.85) compared to European (R^2^ = 0.82) and Sāmoan men (R^2^ = 0.81). Similar trends were observed between Māori (R^2^ = 0.92), European (R^2^ = 0.89), and Sāmoan women (R^2^ = 0.88).

Another New Zealand study compared anthropometric indices to subcutaneous adipose tissue, visceral adipose tissue, and total abdominal fat volumes estimated by MRI [42]. Age- and sex-adjusted models revealed that WC explained 56.9% of the variance in visceral adiposity, 61.2% of the variance in subcutaneous adiposity, and 74.6% of the variance in total abdominal fat volume in the Māori/PI group. WC was also a better predictor of subcutaneous adiposity, visceral adiposity, and total abdominal fat in the Māori/PI group than in the Caucasian group. BMI demonstrated slightly better predictive capacity, explaining 57.9% of variance in visceral adipose tissue, 86.9% of variance in subcutaneous adipose tissue, and 87.5% of variance in total abdominal fat in the Māori/PI group, with lower predictive capacities in the Caucasian group. In contrast, a separate New Zealand study reported that age- and sex-adjusted regression models between BMI and VAT were significant for all visceral adipose tissue compartments in the European group, significant for specific regions in Māori/PI, and nonsignificant for all regions in the Asian cohort [43]. Waist circumference and waist-to-height ratio were not significantly associated with visceral adiposity in European, Māori/PI, or Asian groups.

These indices are also varied in effectiveness as predictors of metabolic health outcomes. Waist circumference cutoffs are integrated into the diagnostic criteria for MetS, although its predictive utility for different health risks may vary depending on the method of measurement [44]. Although the waist-to-hip ratio was proposed as a controlled way to estimate abdominal obesity, it is a worse predictor of metabolic health risk than BMI and waist circumference in some populations [45]. Compared to the waist-to-hip ratio, the waist-to-height ratio may be a better predictor for metabolic health risks in some communities [46], but it may overestimate health risks in shorter individuals. WHT.5R was formulated to mitigate this bias, demonstrating improved predictive capacity in some populations [39], but reduced utility in others [47]. A body shape index is an effective predictor of mortality risk in European cohorts [40], though its practical value may be limited in other contexts [48]. The body adiposity index is a generally consistent proxy measure for body fat percentage, but the relationship between body fat percentage and metabolic risk is not universal [41]. The body roundness index may be a more effective predictor for MetS than other anthropometric indices [49]. Still, the body adiposity and body roundness indices were not uniformly effective indicators of obesity-related insulin resistance [50].

To illustrate racial–ethnic differences in the utility of anthropometric indices, their predictive power for various metabolic outcomes in different Caucasian, Asian, and NHPI cohorts are presented in Table 2 as reported receiver operating characteristic (ROC) area under the curve (AUC) values [31,51,52,53,54,55,56]. In this context, ROC curves evaluate the performance of a predictive model by plotting the true-positive detection rate (sensitivity) against the true-negative detection rate (1—specificity) for a binary classifier. Its AUC summarizes the model’s capacity to distinguish between the two classes [57]. An AUC of 1 would indicate perfect discriminative ability, while an AUC of 0.5 would indicate random discrimination or no predictive power. AUC values may not necessarily be comparable between studies due to modeling differences and covariate considerations. Still, AUC values reported by the same study may be used to compare the predictive capabilities of independent variables.

As an indicator of MetS in Caucasians, the waist-to-height ratio was the most effective index in the Polish cohort. In a New Zealand cohort, waist circumference was an equally effective indicator in women and a more effective indicator for men. For Chinese men, BMI was a slightly better predictor for MetS compared to other indices, but waist circumference, waist-to-height ratio, and the body roundness index demonstrated equally moderate predictive capacity for MetS in Chinese women. The waist-to-height ratio was a moderately effective predictor for hypertension in Polish, Tongan, and Chinese cohorts, but this predictive capacity was matched by other indices (except in Polish women). The waist-to-height ratio was also a moderate indicator for dyslipidemia in Polish, Tongan, and Chinese women but not superior to waist circumference (in Caucasian and Tongan cohorts) or body roundness index (in Polish and Chinese cohorts). In men, however, percent body fat was a better predictor of dyslipidemia in the Tongan cohort, and BMI was a slightly better predictor in the Chinese cohort. In Polish men, BMI, waist circumference, waist-to-height ratio, and percent body fat exhibited comparable, moderate predictive capacity for dyslipidemia.

In a Hawai‘i cohort, the waist-to-height ratio was a slightly better predictor of T2DM in Caucasians, though BMI matched this predictive capacity in Caucasian men. The waist-to-height ratio was also better than other indices as a predictor of T2DM in a cohort in China, though the body roundness index was equally effective for Chinese women. In Hawai‘i, BMI was better than other indices as a moderate predictor of T2DM in Japanese Americans and in Native Hawaiian men. In Native Hawaiian women, BMI, waist circumference, and waist-to-height ratio had similar predictive capacities for T2DM. In a Tongan cohort, the waist-to-hip ratio was consistently more effective than BMI, waist circumference, waist-to-height ratio, and body fat percentage as a predictor of T2DM. Overall, a body shape index had the worst predictive performance for MetS, hypertension, dyslipidemia, and T2DM in Polish and Chinese cohorts.

NHPIs are more likely to be classified as obese by conventional standards. However, these standards vary in their effectiveness as indicators of metabolic health status among ethnically diverse populations. Applying conventional BMI cutoffs as thresholds for data stratification may introduce a misclassification bias [58]. In NHPI health research, this bias can also be introduced using generalized thresholds for any obesity index, whether anthropometric or directly measured, especially if obtained based on non-NHPI populations. As continuous variables, unadjusted indices may present similarly skewed representations with other biological factors. It must also be noted that anthropometric indices are not uniformly indicative of adiposity or body composition and should not be communicated as such until those measures are verifiably relevant in a study population.

Misrepresentation in biomedical data is particularly dangerous in understudied populations that face disparate health outcomes, as misinformation will hinder efforts to understand community-specific factors. To mitigate the risk of such biases, the community-specific relationship between accessible obesity measurement data and the outcome of interest should be considered in scientific communication. Additionally, clarifying anthropometric associations with adiposity may recontextualize their relevance in obesity-related outcomes. Further research is necessary to elucidate the role of fat accumulation in metabolic disease etiology in understudied populations. Until then, the variable metabolic relevance of obesity-related measures should be acknowledged in health disparities research, and such transparency in scientific communication may mitigate the risk of misrepresentations of Indigenous health.

## 3. Heterogeneity in Gut Bacterial Study Findings

### 3.1. Obesity-Related Trends in 16S-Based Metataxonomic Research

Compared to metagenomic approaches, such as whole-genome shotgun sequencing, 16S-based metataxonomic sequencing is an accessible strategy for exploratory gut microbiome profiling regarding laboratory application and data analysis [59]. DNA extraction methods [60], 16S hypervariable region target selection [61], and primer design (which may introduce sequencing bias due to preferential amplification) [62] are steps in the next-generation sequencing workflow that can significantly impact metataxonomic findings.

Aside from methodology, regional and racial–ethnic differences influence the composition of the gut microbiome, and, in turn, the conclusions drawn from related research. For example, a microbiota-based predictive model for metabolic disease risk effective in one district in China was unreliable in another district within the same province [63]. Even in a group of individuals from the same region, gut microbiome composition still varies across racial–ethnic groups after controlling for age, sex, dietary habits, and metabolic health status [64,65]. The current body of literature reporting obesity-related trends in specific gut bacteria presents regional and racial–ethnic diversity that complicates its generalizability to understudied populations. Additionally, inconsistent reports in recent literature demonstrate that community-specific research is necessary to determine gut bacterial associations with NHPI health outcomes. Publications from 2019 to 2024 reporting 16S-based gut bacterial metataxonomic trends in obesity were considered for the review of current literature (Table 3). A flowchart of inclusion/exclusion criteria for the present literature search is illustrated in Figure S1. Methods, BMI cutoffs, and cohort descriptions (including available racial–ethnic information) for the studies included in the present review are summarized in Table S1.

Twenty-one studies were included in this review. One study was conducted in two cohorts of South African women from Bushbuckridge (rural) and Soweto (urban) [81]. Of the studies conducted in Asia, five were conducted in various regions in China (including Beijing, Changzhi, Harbin, and Jinan) [66,68,109,119], one was conducted in Thailand [71], and one was conducted in Pakistan [79]. Of the European studies, one took place in Ireland (Mitchelstown, Cork, and Kerry) [93], three were conducted in various communities across Italy (Emilia Romagna region [103], Sardinia [80], and Siracusa, Sicily [73]), and two were conducted in Spain (Navarra [85] and Madrid [88]). Study populations were predominantly women for the Navarra [85], Emilia Romagna [103], and Sardinia studies [80], but predominantly men for the studies conducted in Siracusa [73]. From North America, five studies were conducted in the US (Boston, MA [67]; Nashville, TN [98]; Los Angeles, CA [84]; Davis, CA [107], and a multi-center study across Chicago, IL, Miami, FL, Bronx, NY, and San Diego, CA [91]). One was conducted in Guadalajara, Mexico [90]. One study from the Pacific region was conducted in a group of men from Auckland, New Zealand [104], and one was conducted in an NHPI-enriched cohort on Oʻahu, Hawai‘i [72].

Among the significant results presented by these studies, consistently positive associations with obesity were reported for four genera: *Catenibacterium*, *Megasphaera*, *Allisonella*, and *Streptococcus*, each representing the Bacillota (Firmicutes) phylum. *Catenibacterium* was associated with obesity in cohorts from Bushbuckridge (but not Soweto) [81], Madrid [88], and the multi-center study in Hispanic US residents [91]. *Megasphaera* was associated with obesity in one Italian [80] and three United States studies [84,91,98], cohorts that tended to have a higher representation of women and individuals from Caucasian and Hispanic backgrounds. *Allisonella* was associated with obesity in cohorts from Spain [85], the US [98], Mexico [90], and Pakistan [79]. *Streptococcus* was associated with obesity in cohorts from Soweto (but not Bushbuckridge) [81], Sardinia [80], and various regions in the US [84,91]. Notably, the cohorts in which these trends were observed had a relatively low representation among Asian groups.

Consistently negative associations with obesity were reported for four genera: *Bifidobacterium*, *Barnesiella*, *Anaerostipes*, and *Coprococcus*. These trends were observed in a diverse set of study populations, including cohorts from China [66], Thailand [71], New Zealand [104], Hawai‘i [72], Mexico [90], Spain [88], Ireland [93], and Italy [74,104]. While these reports may seem widespread, significant trends were only represented in a subset of cohorts from each region. *Bifidobacterium*, *Barnesiella*, and *Coprococcus* were significantly associated with obesity in one cohort from Jinan and Beijing [66] but not from cohorts from Harbin or Changzhi [68,109]. *Bifidobacterium* and *Coprococcus* were highlighted in Siracusa [73] and Emilia Romagna [103] but not in Sardinia [80]. *Barnesiella* was associated with obesity in the Madrid cohort [88] but not in the Caucasian cohort from Navarra, Spain [85]. Interestingly, *Bifidobacterium* was previously characterized as a “lean-associated” genus in Eastern populations [120], a trend not consistently reported in the current literature.

Significant study findings in the current literature also exhibited a high degree of heterogeneity. *Bacteroides* was positively associated with obesity in a Caucasian cohort in Navarra [85] but negatively associated with obesity in Bangkok [71] and Los Angeles [84]. Although *Bacteroides* is conventionally associated with improved metabolic outcomes [85], its association with obesity was only observed in three studies in the present review.

*Prevotella* was positively associated with obesity in China (Jinan and Beijing [66]), Thailand [71], both cohorts from South Africa [81], and in multiple cities across the US (Los Angeles, Chicago, Miami, Bronx, and San Diego) [84,91]. However, *Prevotella* was not significantly associated with obesity in other studies conducted in China (Beijing [119] or Changzhi [109]), the US (Boston [67], Nashville [98], or Davis [107]), or in any of the studies conducted in Europe or the Pacific Islands. *Prevotella* has previously been described as obesity-associated in the West but lean-associated in the East [120], but regional trends were heterogeneous among recent study findings. *Prevotella* may conditionally ameliorate insulin resistance depending on dietary patterns [92], and community-specific factors may contribute to these inconsistencies.

The association between *Faecalibacterium* and obesity was positive in Harbin [68] but reportedly negative otherwise, as seen in Jinan and Beijing [66], Boston [67], Hawai‘i [72], and Navarra [85]. The tendency toward a negative relationship with obesity is consistent with previous descriptions of *Faecalibacterium* as a potential mediator of fat absorption and meta-inflammation [63,98]. However, significant trends were directionally heterogeneous and only observed in a subset of the studies conducted in Asia, Europe, and the US.

*Ruminococcus* was positively associated with obesity in South Africa [81] and New Zealand [104]. More frequently, *Ruminococcus* was negatively associated with obesity. Significantly negative trends were reported by studies conducted in China [66], Thailand [71], Pakistan [79], Italy [103], and the US [91]. In a meta-inflammatory context, these negative trends are consistent with previous characterizations of *Ruminococcus* as a producer of beneficial short-chain fatty acids (SCFAs) associated with attenuated MetS symptoms [16,98]. However, individuals with obesity tend to have higher total fecal concentrations of SCFAs, a tendency that *Ruminococcus* may exacerbate [101,102].

In contrast, *Megamonas* was most frequently reported to have a positive trend in obesity. This association was observed in cohorts from Jinan and Beijing [66], Changzhi [109], Navarra [85], Los Angeles [84], and Sardinia [80], consistent with previous reports of increased production of proinflammatory biomarkers and MetS risk with its growing prevalence [110]. However, one study from Harbin reported a negative relationship between *Megamonas* and obesity [68].

Although each of the studies included in the present review employed 16S-based metataxonomic techniques, their definitions of obesity tended to vary (Table S1). Conventional or adjusted anthropometric cutoffs were sometimes used for data stratification. It was similarly common for some studies to treat various obesity measures as covariates in regression or other multivariate analyses. Even after adjusting for the metabolic relevance of obesity measures within respective study cohorts, the heterogeneity among study findings suggests that the gut microbiome is differentially associated with health on a community-specific basis. Therefore, due to the highly context-dependent nature of the microbiome and the underrepresentation of minority populations in biomedical research, gut bacterial associations with metabolic outcomes are largely uncharacterized for NHPI communities.

### 3.2. NHPI-Inclusive Literature Presents Community-Specific Trends

In 1974, Moore and Holdeman characterized gut microbiome composition in a cohort of 20 Japanese Hawaiian men aged 60–80 years [121]. Fecal bacteria were isolated, cultured, and identified using techniques described in the Anaerobe Laboratory Manual [122]. This study resulted in the discovery and first description of the *Coprococcus* genus [123], which has since been recognized for its probiotic potential in ameliorating obesity, gastrointestinal disorders, and meta-inflammation [124].

Articles published between 2014 and 2024 were considered for the present literature review regarding NHPI-specific characterizations of the gut microbiome. A flowchart of inclusion/exclusion criteria for the present literature search is illustrated in Figure S2. Methods and cohort descriptions are summarized for each study in Table S2. Five selected studies were included in Table 4. One study measured gut bacterial associations with hepatic adiposity in multiethnic cohort (MEC) study participants from Hawai‘i and Nevada [125]. One study was a methodological evaluation of SparseMCMM_HD as an analytical tool for causal mediation between gut bacteria and BMI disparity [126]. Two studies were conducted in NHPI-enriched communities in Hawai‘i [72,127]. One study compared the prevalence of pathogenic bacteria in two Torres Strait Islands, Waiben and Mer [128].

In the recent MEC study, the non-alcoholic fatty liver disease (NAFLD) state was designated at liver fat > 5.5%, measured by abdominal MRI [125]. Beta-binomial regression models were then used to estimate associations between the prevalence of specific gut bacteria and the NAFLD state. Obesity is a major risk factor for NAFLD due to its influence on fat accumulation, lipid metabolism, and meta-inflammation, which can cause hepatic cell injury and further metabolic dysfunction [129].

In Native Hawaiians, NAFLD is associated positively with gut bacterial genera *Parasutterella* and *Megamonas* [125]. *Megamonas* was frequently associated with obesity, meta-inflammation, and MetS in other populations [110]. However, the metabolic impact of *Parasutterella* may not necessarily be discerned through measures of its abundance [130]. *Parasutterella* may be a significant succinate producer, which plays a complex role in functional and dysfunctional metabolism [130,131]. In fact, *Parasutterella* was negatively associated with obesity in a Madrid cohort [88], demonstrating intergroup differences in its functional relevance. A positive association between *Lactobacillus* and NAFLD was observed for Native Hawaiian and African American groups [125]. While this contradicts its presumed role in alleviating metabolic disorders via meta-inflammatory modulation [132], *Lactobacillus* has also been identified as an obesity-associated genus in Western populations [120].

In Native Hawaiians only, NAFLD is associated negatively with *Idiomarina*, *Klebsiella*, *Negativibacillus*, and *Intestinimonas*. *Idiomarina* is characterized by its extremophilic strains isolated from deep-sea sediment and submarine volcanoes [133,134]. Some members of *Klebsiella* have been implicated as causal determinants of gastrointestinal diseases [135]. In a meta-inflammatory context, this implication is inconsistent with its reduced prevalence in Hawaiians with NAFLD. *Negativibacillus* is enriched by consuming ultra-processed foods, which is linked to obesity, dyslipidemia, dysglycemia, and CVD [136]. This link makes its negative association with NAFLD in Native Hawaiians unexpected. The opposite trend was observed in White individuals with NAFLD from the same study, suggesting racial–ethnic differences in the metabolic impact of *Negativibacillus*. *Intestinimonas* may be protective against diet-induced dysbiosis as a major butyrate producer in lysine-rich environments [137]. Its negative association with NAFLD may indicate that butyrate is especially relevant to metabolic outcomes in Native Hawaiians compared to other racial–ethnic groups.

The MEC study also used a whole-genome shotgun metagenomic sequencing approach to assess gut bacterial metabolic capacity [125]. In Native Hawaiian women, butyrate kinase (BUK) was inversely associated with percent liver fat. Consistent with these findings, BUK gene expression was negatively correlated with A1c test results in the NHPI-enriched cohort [72]. While a high-fiber diet is typically considered butyrogenic [138], proportional dietary vegetable intake did not significantly influence BUK gene abundance or expression. In the same NHPI-enriched cohort, one study found that *Akkermansia* was negatively correlated with BUK gene abundance and expression [73], and another found that *Akkermansia* correlated negatively with self-esteem [128]. While butyrogenic pathways may be metabolically relevant for NHPI health and well-being in this population, further research is necessary to elucidate direct connections between butyrate production and health outcomes.

One of the papers cited above was a methodological evaluation of SparseMCMM_HD as an analytical tool for causal mediation between gut bacteria and BMI disparity [126]. Data obtained from the American Gut Project (AGP) were used to analyze disparities between Caucasian and Asian and Pacific Islander (API) populations. In this study, Caucasians had a significantly higher BMI than APIs. Additionally, the proportion of Asian to NHPI representation in this API group is unclear. While disaggregated findings are not reported in this publication, SparseMCMM_HD may be a valuable tool for assessing gut bacterial mediation in the context of NHPI health disparities.

Our two recent NHPI-inclusive studies were conducted in NHPI-enriched cohorts on Oʻahu, Hawai‘i [72,127]. In one of these studies, we reported that *Bifidobacterium* had a moderately negative correlation with age and slightly less prominent correlations in the same direction with BMI and A1c levels. This was the only taxon simultaneously associated with A1c and BMI. While this trend may suggest that *Bifidobacterium* plays a protective role in obesity-related health risks, further investigation is necessary to determine whether this trend is still observed after age adjustment. These results additionally suggest that BMI is not a particularly effective predictor of T2DM within the NHPI population. In this instance, significant BMI-related results may not be informative about obesity-related T2DM risk. However, these results may be recontextualized if BMI is verified as an effective predictor for other health outcomes in this population.

In contrast, three taxa exhibited a consistently positive correlation with BMI, A1c levels, and age: Deferribacteres (Deferribacterota), *Mucispirillum*, and *Shigella*, which were not frequently associated with obesity among other populations (Table 3). Since age may disproportionately influence cardiometabolic risk, especially within the NHPI population compared to other racial–ethnic groups [7], these taxa may mediate the age-related presentation of disparate health outcomes in a community-specific manner. *Lactococcus* also correlated positively with the A1c score but not with age or BMI, although further research is necessary to uncover its metabolic relevance in this community. *Prevotella* was negatively correlated with age but was not highlighted for significant associations with other risk factors among the NHPI-inclusive cohort studies.

The study conducted on the Torres Strait Islands found that individuals from Mer had a significantly higher risk for cardiometabolic disease compared to individuals from Waiben, even after adjusting for age [128]. This increased risk was unexpected. The Waiben population tended to have a more Westernized diet and lower levels of physical activity, and there was no significant difference in vegetable consumption, alcohol/smoking, or BMI between the islands. *Proteobacteria* and *Euryarchaeota* were significantly more abundant in Mer than in Waiben, which may suggest their involvement in adverse health outcomes. This study also found that *Lachnospiraceae bacterium 8_1_57FAA* causally mediated the positive trend in mean arterial pressure with increasing intake of sugar-sweetened beverages after age and site adjustment.

Gut bacterial associations with health outcomes may be conditional and community-specific. NHPI-inclusive gut microbiome research presents distinct trends from those obtained in other studies. Further research is necessary to elucidate their metabolic relevance in these populations. Even among NHPI-inclusive research, gut bacterial health impacts are highly variable between distinct populations. Therefore, community- and individual-specific research is required for an accurate characterization of metabolically relevant metataxonomic trends. However, NHPI-inclusive data tend to be aggregated, and NHPIs remain underrepresented in health disparities research [139]. NHPI-inclusive research would enable a relevant and accurate understanding of the gut bacterial impact on metabolic disease etiology in these populations that accounts for the environmental context, laying solid groundwork for advancing health equity.

## 4. Considerations for Community Context-Specific Research

Anthropometric indices are widely used in obesity assessment due to their simplicity and cost-effectiveness, offering a practical and easily accessible approach for large-scale studies and clinical settings. However, they are not universally effective indicators of metabolic health, and they should not be communicated as such. In the context of metabolic research, conventional or generalized standards for obesity may introduce misclassification biases, especially for underrepresented populations [58].

Applications of conventional obesity measures in biomedical research are still informative, as they are commonly used in real-world healthcare decisions. However, their variable intergroup utility as predictors of metabolic outcomes should be acknowledged in research dissemination to prevent misleading characterizations of Indigenous health. The same acknowledgment should extend to the variable relationship between anthropometric indices and adiposity. Further investigation is necessary to understand relationships among anthropometrics, adiposity, and metabolic outcomes, especially in understudied Indigenous populations, such as NHPIs. Until then, researchers should exercise caution when presenting data measured by differentially effective risk assessment tools and when communicating the implications of their study findings.

The current body of literature regarding 16S-based gut metataxonomics presents a broad range of heterogeneity not only in research findings but also in the diversity among study populations. Inconsistencies in current literature demonstrate that gut bacterial associations with obesity-related outcomes may be community-specific. Therefore, the generalizability of gut microbiome research is limited, even across NHPI populations. NHPI-inclusive studies with disaggregated data may provide a clearer understanding of metabolic health in these communities, enabling the development of tailored strategies to address their specific needs.

NHPIs remain underrepresented in current literature, which may be due to exclusion, data aggregation [139], or the underestimated significance of self-reported racial–ethnic data collection. Self-reported race/ethnicity may reflect patterns in shared lifestyle, behavioral, and cultural experiences among members of the same community [65], influences that are not explained by genetic ancestry alone. Given the impact of self-identified race/ethnicity, cultural considerations must be considered at every step of the scientific research process.

While health disparities research is generally well intentioned, the process of designing, implementing, and interpreting research data requires a deeper understanding of cultural context if the intent is for research studies to provide understandable and relevant meaning to the communities it aims to impact and serve [140]. Limited conclusions of Indigenous data may hinder the development of strategies for addressing community-specific factors and stymie community efforts in building a body of knowledge about themselves while engaging in scientific research to better understand the social/environmental context relevant to health.

## 5. Conclusions

Although anthropometric indices can be applied as easily accessible health risk assessment tools for obesity-related outcomes, their utility and metabolic relevance may vary among ethnically diverse populations. Namely, body mass index (BMI), which is conventionally used in clinical and research settings to diagnose obesity, may differentially associate with adiposity and metabolic risk between racial–ethnic groups. Community-specific research is necessary to accurately contextualize anthropometric associations with adiposity and obesity-related disease etiology among understudied and health-disparate populations, including Native Hawaiians and other Pacific Islanders (NHPIs).

The gut microbiome is an area of particular research interest as a mediator of obesity-related complications, which may be a promising avenue for addressing community-level disparities. However, the heterogeneity among reports from recent 16S-based metataxonomic studies demonstrates that gut bacterial associations with obesity may vary with regional, racial–ethnic, and other contextual factors. Therefore, the generalizability of obesity-related gut microbiome research is limited.

Due to the employment of a nonsystematic approach to current literature, the present review is inherently limited. Moreover, place names were not included as search terms to limit the number of irrelevant returns, which may have resulted in the exclusion of otherwise relevant, NHPI-inclusive literature.

Among the literature pertaining to the gut microbiome included in the present review, nonsignificant results were inconsistently reported—they were sometimes featured in the main text, in the supplemental materials, or excluded entirely. With this, nonsignificant reports were excluded from Table 3 and Table 4. The inclusion of nonsignificant trends may have complemented the present review by further highlighting the heterogeneity among recent study findings. The heterogeneity in analytical approaches among the studies included for review, namely, those cited in Table 3, is another considerable limitation. Main research questions and hypotheses varied between studies, and methodological/analytical approaches varied accordingly (Table S1). Therefore, conclusions about gut bacterial trends in obesity may not necessarily be straightforward or uniform among the studies included for review. Moreover, some studies included in the present review used BMI to designate obesity status, and subsequently, for data stratification, which may not consistently reflect adiposity or metabolic health risk between distinct study populations.

Nonetheless, such heterogeneity highlights challenges in the generalizability of gut microbiome research, especially of understudied populations. For future strategies leveraging host–microbe interactions to address obesity-related disparities, NHPI-inclusive research is required to elucidate racial–ethnic differences in metabolic disease risk. Furthermore, additional research is necessary to characterize the relationship between anthropometric indices, adiposity, and metabolic risk in NHPI populations. This characterization may ensure accurate representations of Indigenous health in any other obesity-related research, which may be valuable to the development of novel strategies for addressing NHPI health disparities.

## Figures and Tables

**Table 1:** Body mass index (BMI) thresholds (inclusive lower bounds) to designate overweight and obesity status in different regional and racial–ethnic communities.

Ref.	Overweight	Obesity	Community	Location	Institution
[25]	25	30	^A^	International	World Health Organization (WHO)
[26]	23	25	Asian ^A^	Asia-Pacific Region	International Diabetes Institute
	26	32	Polynesian ^B^		
[27]	24	28	Chinese ^A^	China	The Working Group on Obesity in China
[28]	23	25	Japanese	Japan	Japan Society for the Study of Obesity
[28]	23	25	Korean	South Korea	Korean Society for the Study of Obesity
[29]	23	25	Asian Indian ^B^	India	Indian Consensus Group
[22]	19.2	23.9	South Asian ^A^	England	Primary Care Diabetes Society; Nuffield Department of Population Health, University of Oxford
	22.1	26.6	Arab ^A^		
	22.2	26.9	Chinese ^A^		
	23.4	28.1	Black ^A^		
	25	30	White ^A^		
[23]	25	30	Chinese ^B^	China	World Health Organization (WHO) Expert Consultation
	22	27	Chinese ^B^	(Hong Kong)	
	22	27	Indonesian ^B^	Indonesia	
	24	29	Japanese ^B^	Japan	
	23	27	Singaporean ^B^	Singapore	
	23	28	Thai ^B^	Thailand (urban)	
	25	30	Thai ^B^	Thailand (rural)	

^A^ Determined using community-specific metabolic risk factors. ^B^ Determined using body fat percentage equivalents at conventional BMI cutoffs in White populations.

**Table 2:** Sex-specific receiver operating characteristic area under the curve (ROC-AUC) values for anthropometric indices in predictive models for cardiometabolic outcomes in Caucasian, Asian, and NHPI communities. Cells are left blank for unreported AUC values.

Ref.	Ethnicity	Men							Women						
		BMI	WC	WHR	WHtR	%BF	ABSI	BRI	BMI	WC	WHR	WHtR	%BF	ABSI	BRI
**Metabolic syndrome (MetS)**															
[51]	Caucasian	0.75			**0.76**	0.74	0.60	0.73	0.73			**0.76**	0.72	0.64	0.75
[31]	Caucasian	0.78	**0.85**		0.81				0.77	**0.84**		**0.84**			
	Māori	0.81	**0.84**		0.82				0.77	**0.81**		**0.81**			
[52]	Chinese	**0.72**	0.71	0.67	0.71		0.57	0.71	0.69	**0.70**	0.66	**0.70**		0.59	**0.70**
**Hypertension**															
[53]	Caucasian	**0.66**	**0.66**		**0.66**	0.65	0.54	0.64	0.68	0.68		**0.69**	0.68	0.58	0.68
[54]	Tongan	0.67	**0.68**	0.64	**0.68**	0.61			0.71	**0.72**	0.62	**0.72**	0.64		
[52]	Chinese	0.64	0.66	0.64	**0.67**		0.60	0.67	0.67	0.70	0.66	**0.71**		0.63	**0.71**
**Dyslipidemia**															
[53]	Caucasian	0.64	0.64		0.64	0.64	0.54	0.62	0.64	0.66		0.66	0.64	0.61	0.66
[55]	Tongan	0.70	0.72	0.69	0.71	**0.75**			0.60	0.66	0.65	0.66	0.55		
[52]	Chinese	**0.69**	0.68	0.65	0.67		0.55	0.67	0.66	0.67	0.65	**0.68**		0.58	**0.68**
**Type 2 diabetes mellitus (T2DM)**															
[56]	Caucasian	**0.75**	0.74	0.71	**0.75**				0.78	0.78	0.75	**0.79**			
	Hawaiian	**0.70**	0.69	0.67	0.69				**0.72**	**0.72**	0.69	**0.72**			
	Japanese	**0.68**	0.66	0.64	0.66				**0.72**	0.71	0.69	0.71			
[55]	Tongan	0.67	0.72	0.75	0.73	0.71			0.63	0.69	**0.72**	0.70	0.54		
[52]	Chinese	0.66	0.70	0.69	**0.71**		0.60	0.67	0.67	0.70	0.66	**0.71**		0.63	**0.71**

BMI = body mass index. WC = waist circumference. WHR = waist-to-hip ratio. WHtR = waist-to-height ratio. %BF = percent body fat (measured by dual-energy X-ray absorptiometry). ABSI = a body shape index. BRI = body roundness index. The highest receiver operating characteristic area under the curve (ROC-AUC) values for men and women from each study are bolded. Covariate adjustments may vary between studies.

**Table 3:** Obesity-related prevalence trends in specific gut bacteria from recent 16S-based metataxonomic studies. Taxa are listed at phylum and genus levels. Genera are only listed here if they are significantly associated with obesity in at least three studies in the literature review.

	Trends in Obesity		Metabolic Relevance
	Negative	Positive	
Actinomycetota ^A^	[66,67]	[68]	Produces fungicidal and antibiotic metabolites [69,70].
*Bifidobacterium*	[66,71,72,73]		Attenuates LPS-induced meta-inflammation, insulin resistance [74,75], visceral fat, plasma triglycerides [76]; SCFA producer [77], variable impact on fecal SCFA [78].
Bacteroidota ^B^	[68,79,80]	[66,67,73,81]	Can produce H_2_S from host mucosal glycans, which causes epithelial damage [82,83].
*Bacteroides*	[71,84]	[85]	Inhibits lipid accumulation in preadipocytes; alleviates HFD-induced obesity. Restores glucose/lipid homeostasis [86]. SCFA producer [77]; increased fecal acetate [78].
*Parabacteroides*	[66,68,80]	[81,84] ^H^	Facilitates the production of secondary bile acids (lipid homeostasis/insulin signaling), activates intestinal gluconeogenesis, and alleviates obesity [87].
*Alistipes*	[66,88]	[81] ^H^	Alleviates inflammation and atherosclerotic CVD [89].
*Prevotella*	[68,79,90]	[66,71,81,84,91]	Stimulates proinflammatory cytokine production [84]; Negatively correlated with intestinal permeability [78]; glycemic control in high-fiber diet but not HFD [92].
*Barnesiella*	[66,88,93]		Produces SCFAs involved in glucose/energy homeostasis; mediates inflammation [94].
Bacillota ^C^	[66,67,73]	[68,79,80]	Enables higher calorie absorption from dietary nutrients, promoting weight gain [95].
*Phascolarctobacterium* ^D^	[93]	[81,91] ^H^	Propionate/acetate producer; expanded in HFD-induced obesity [96]; negatively correlated with stool SCFA concentrations [78].
*Catenibacterium*		[81,88,91] ^H^	Enriched in Western diet; more prevalent in metabolically unhealthy obesity than in metabolically healthy obesity [97].
*Megasphaera*		[80,84,91,98]	Can produce butyrate from glutamate and lysine. Produces ammonia as a byproduct [99].
*Allisonella*		[79,85,90,98]	Produces histamine, meta-inflammatory [100]
*Oscillibacter*	[66,88]	[81]	Enriched in HFD [94]; facilitates diet-induced obesity and inflammation [101].
*Faecalibacterium*	[66,67,72,85]	[68]	Butyrate-producing; bile-hydrolyzing, which regulates fat absorption and glucose/lipid homeostasis [67]. Inhibits IL-8 and IL-6 production; induces IL-10 production in PBMCs [102].
*Ruminococcus*	[66,71,79,91,103]	[81,104]	Attenuated MetS symptoms [16]; positively associated with Crohn’s [105]; induces TNF production [106]; SCFA producer [77].
*Sporobacter*	[88]	[81,98] ^I^	Negatively correlated with fecal concentrations of butyrate and acetate [78].
*Clostridium*	[71]	[90,107]	Contains the highest number of bacterial strains with butyrate production capacity [108] but is not significantly correlated to fecal SCFA concentrations [78].
*Clostridium XIVa*	[66,93]	[81,88] ^I^	
*Clostridium IV*	[66,93]	[81,93] ^H^	
*Megamonas*	[68]	[66,80,84,85,109]	Positively correlated with proinflammatory biomarkers and increased risk for MetS [110].
*Romboutsia*	[88]	[90,109]	Positively associated with fecal SCFA concentrations [78].
*Anaerostipes*	[90,93,104]		Produced propionate and acetate from inositol, which alleviated insulin resistance in HFD-fed mice [111].
*Blautia*	[90]	[66,68]	Induces pro- and anti-inflammatory responses; varies at the species level [112].
*Coprococcus*	[66,93,103,104]		May attenuate intestinal permeability [78].
*Roseburia*	[103]	[68,90]	SCFA producer [77]; positively correlated with fecal SCFA concentrations [78]. Primary degrader of β-mannans [113].
*Streptococcus*		[80,81,84,91] ^I^	Correlated with intestinal inflammation [78].
Pseudomonadota ^E^	[79]	[67,68,73]	May induce meta-inflammation via LPS [114].
*Haemophilus*	[85]	[71,81,84] ^I^	Associated with intestinal inflammation [115].
Fusobacteriota ^F^	[68,88]	[66,72]	Inhibits inflammation in colorectal cancer [116].
Verrucomicrobiota ^G^	[73,79,88]	[68]	Major mucin-degrading bacteria; associated with upregulated expression of anti-inflammatory cytokines and Tregs [117]; promotes intestinal barrier integrity [118]; *Akkermansia* negatively correlated with fecal acetate and butyrate concentrations [78].

^A^ Actinobacteria. ^B^ Bacteroidetes. ^C^ Firmicutes. ^D^ Phascolarctobacterium. ^E^ Proteobacteria. ^F^ Fusobacteria. ^G^ Verrucomicrobia. ^H^ Significant results in Bushbuckridge and not Soweto [82]. ^I^ Significant results in Soweto and not Bushbuckridge [81]. LPS = lipopolysaccharide; endotoxin. SCFA = short-chain fatty acids. HFD = high-fat diet. CVD = cardiovascular disease. IL = interleukin. TNF = tumor necrosis factor. PBMCs = peripheral blood mononuclear cells. MetS = metabolic syndrome. Tregs = regulatory T cells. For each gut bacterial taxon, studies that reported a significant positive or negative association with obesity are listed in the “trends in obesity” columns. Positive trends were designated for specific taxa if they were more prevalent in obesity compared to non-obese groups or positively associated with obesity metrics in a regression analysis. Negative trends were designated for specific taxa if they were less prevalent in obesity compared to non-obese groups or negatively associated with obesity metrics in a regression analysis. Baseline trends were prioritized for qualifying intervention studies.

**Table 4:** Significant metataxonomic trends in NHPI-inclusive research.

Ref.	Community	Gut Bacterial Taxa	Metataxonomic Trends
[125]	MEC Study Native Hawaiians from Honolulu, HI or Los Angeles, CA	*Parasutterella* *	Positively associated with NAFLD (liver fat > 5.5%)
		*Megamonas* *	
		*Lactobacillus*	
		*Idiomarina* *	Negatively associated with NAFLD (liver fat > 5.5%)
		*Klebsiella* *	
		*Negativibacillus* *	
		*Intestinimonas* *	
		*Erysipelatoclostridium*	
		*Clostridium* ^A^	
[126]	US-residing Asian and Pacific Islanders (API)	*A. muciniphila*	Positively mediates BMI disparity
		*B. adolescentis*	Negatively mediates BMI disparity
[72]	NHPI-enriched cohort in Honolulu, HI	Fusobacteria ^B^	Positively correlated with BMI
		Lentisphaerae ^C^	Negatively correlated with BMI
		*Bifidobacterium*	
		Deferribacteres ^D^	Positively correlated with A1c score
		*Lactococcus*	
		*Mucispirillum*	
		*Shigella*	
		Bacteroidetes ^E^	Negatively correlated with A1c score
		*Bifidobacterium*	
		*Faecalibacterium*	
		Deferribacteres ^D^	Positively correlated with age
		Proteobacteria ^F^	
		*Mucispirillum*	
		*Shigella*	
		Actinobacteria ^G^	Negatively correlated with age
		Bacteroidetes ^E^	
		*Bifidobacterium*	
		*Prevotella*	
		*B. adolescentis*	
[127]	NHPI-enriched cohort in Honolulu, HI	*Mitsuokella*	Positively correlated with self-esteem
		*Collinsella*	
		*Megasphaera*	
		*Herbaspirillum*	Negatively correlated with self-esteem
		*Akkermansia*	
		*Lachnoclostridium*	
		*A. muciniphila*	
[128]	Torres Strait Islands (Waiben and Mer)	Proteobacteria ^F^ Euryarchaeota	More prevalent in Mer
		*Lachnospiraceae bacterium 8_1_57FAA*	Causally mediates the association between sugar-sweetened beverage intake and mean arterial pressure ^H^

* Uniquely observed in Native Hawaiian and Other Pacific Islander (NHPI) groups compared to other groups in the study. ^A^ *Clostridium sensu stricto 1*. ^B^ Fusobacteriota. ^C^ Lentisphaerota. ^D^ Deferribacterota. ^E^ Bacteroidota. ^F^ Pseudomonadota. ^G^ Actinomycetota. ^H^ Determined using average causal mediation effect (ACME). NAFLD = non-alcoholic fatty liver disease. MEC = multiethnic cohort. API = Asian and Pacific Islanders. Species were only listed if they were mentioned in at least one other paper included for review.

## Supplementary Materials

The following supporting information can be downloaded at: https://www.mdpi.com/article/10.3390/nu16244268/s1. Figure S1: A flowchart of inclusion/exclusion for the (non-comprehensive) review of current literature is presented in Table 3. Table S1: Methodological summary for recent studies on obesity-related trends in 16S-based metataxonomics. Figure S2: Flowchart of inclusion/exclusion for the (non-comprehensive) review of current literature using the PubMed database. Table S2: Methodological summary for recent studies on obesity-related trends in 16S-based metataxonomics.
