# Supplementary material for: Racial–Ethnic Disparities of Obesity Require Community Context-Specific Biomedical Research for Native Hawaiians and Other Pacific Islanders
Source: Nutrients. 2024 Dec 11;16(24):4268. doi: 10.3390/nu16244268 (PMC11676216; doi:10.3390/nu16244268)
Supplement: Supplementary file 1 [file nutrients-16-04268-s001.zip › nutrients-3364111-supplementary.pdf]

nutrients

MDPI

Supplementary Materials

Filters applied: Free full text, in the last 5 years, English, Human subjects, Exclude preprints.

Excluded: reviews, systematic reviews, neonatal/infants/children or pregnant women only.

Search Query: 16s AND (obese OR obesity OR fat) AND (stool OR gut OR fecal OR faecal OR colonic) NOT ((Review[Publication Type]) OR (Systematic Review[Publication Type])) NOT (child[Title] OR children[Title] OR infant[Title] OR infancy[Title] OR childhood[Title] OR infants[Title] OR neonatal[Title] OR neonates[Title] OR offspring[Title]) NOT (pregnant[Title] OR pregnancy[Title] OR

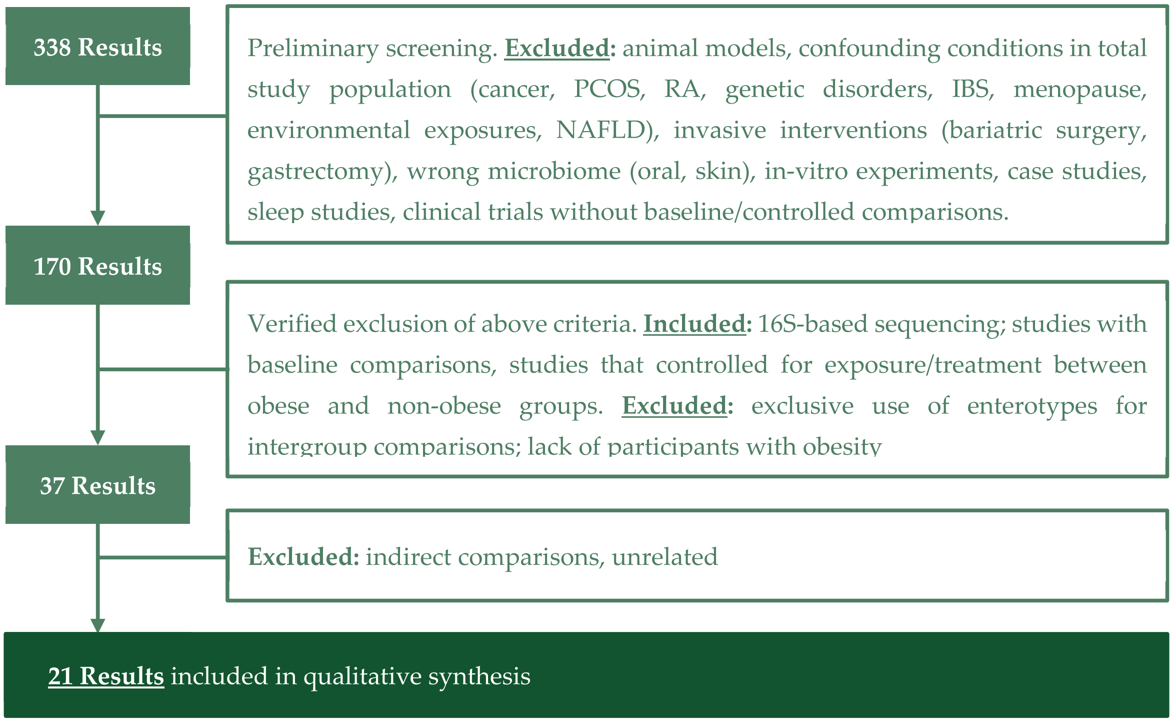

Figure S1. A flowchart of inclusion/exclusion for the (non-comprehensive) review of current literature is presented in **Table 3**. Studies listed in the "metabolic relevance" column were not subject to the same inclusion/exclusion criteria. This search was conducted using the PubMed database. Interventions/clinical trials were included if they presented baseline/treatment-adjusted results.

Table S1. Methodological summary for recent studies on obesity-related trends in 16S-based metataxonomics (2019-2024).

	Obesity group stratification			Summary of study methodology			Summary of study population
**Ref**	**Group**	**Criteria**	**N**	**hvr**	**Location**	**Analysis**	
[67]	MHNO		60	V4	Boston, MA	Wilcoxon Rank-Sum	91% Caucasian (N=20), 9% other (N=2). 91% female (N=20), 9% male (N=2). Excluded: T2DM, NASH, MetS, weight loss surgery, dysphagia, immune deficiencies, antibiotic/probiotic use.
	MHOB	BMI ≥35	22				
[85]	MHNO	BMI ≤ 25	25	V3-V4	Navarra, Spain	Student's t-test	Caucasians. Excluded: DM, CVD, hypertension, pregnant/lactating, obesity-related medications.
	MHOB	BMI 30-40	78				
[91]	NO	BMI 18.5-25	293	V4	Chicago, IL; Miami, FL; Bronx, NY; San Diego, CA	Multiple linear regression (adjusted for age, sex, field center, and Hispanic background)	Hispanic US residents have Mexican or Mexican American, Puerto Rican, Cuban, Central, and South American backgrounds. 1st, "1.5th", and 2nd generation immigrants.
	OW	BMI 25-30	652				
	OB I	BMI 30-35	458				
	OB II	BMI 35-40	176				
	OB III	BMI > 40	103				
[80]	MHNO	BMI 18.5-25	46	V3-V4	Sardinia, Italy	LEfSe	Subjects of Sardinian origin. Of OB individuals, N=30 had MetS, and N=16 did not.
	OB	BMI ≥ 25	46				
[71]	MHNO		9	V3-V5	Bangkok, Thailand	LEfSe; Kruskal-Wallis & Wilcoxon Rank-Sum	Thai participants aged 24–43 years.
	MUOB	BMI > 30; MetS	11				
[80]	NO	BMI < 25	30	V3 –V4	Bushbuckridge and Soweto, South Africa	Kruskal-Wallis & Wilcoxon Rank-Sum	HIV-negative African women from Bushbuckridge (rural; N=119) and Soweto (urban; N=51), South Africa.
	OW	BMI 25-30	31				
	OB	BMI ≥ 30	106				
[93]	MHNO	BMI ≤ 30	191	V3-V4	Mitchelstown, Cork, and Kerry, Ireland	PERMANOVA; DESeq2 (age, sex, diet,	N=382, aged 69.9±5 years, 50.79% male. Excluded: antibiotic use (4 weeks).

	MUNO	BMI ≤ 30; MetS	61			alcohol, smoking, T2DM, CVD, medication adjusted)	
	MHOB	BMI > 30	66				
	MUOB	BMI > 30; MetS	64				
[79]	MHNO	BMI < 25	20	V3-V4	Pakistan	Kruskal-Wallis Rank-Sum	Adults aged 25–55 years, belonging to the ethnic Punjabi population of Pakistan. Excluded: GI complications, antibiotic use (3 months)
	MUOB	BMI > 30; T2DM	40				
[109]	MHNO	BMI < 24	20	V4	Changzhi, China	LEfSe	T2DM patients (disease duration ≤5 years; N=30 cases for OB, N=30 cases for NO). Excluded: T1DM; recent surgery; antibiotic, probiotic use; cancer; pregnancy, lactation; weight-affecting drug use; infectious disease; mental, endocrine, GI, liver, kidney, cardiopulmonary conditions.
	MUNO	BMI < 24; T2DM	30				
	MUOB	BMI ≥ 28; T2DM	30				
[88]	MHNO	BMI 18-25	13	V3–V4	Madrid, Spain	LEfSe	NO: 7F/6M. OB: 7F/6M. Excluded: antibiotic, probiotic use (6 months); metabolic, inflammatory, infectious, autoimmune disease; cancer.
	MHOB	BMI > 30	13				
[104]	NO	BMI 20-25	14	V3-V4	Auckland, New Zealand	LEfSe	N=32 men aged 20-45 years. Non-smokers, sedentary to recreationally active. Excluded: medication, antibiotic, probiotic use (2 months)
	OB	BMI 28-35	15				
[66]	NO	BMI ≤ 25	21	V3-4	(OB) Jinan, Shandong Province, China; (NO) Beijing, China	STAMP	OB: N=21 (16M/5F mean age 35 years) from Jinan, Shandong Province. Excluded: antibiotics (1 month); weight-related drugs. Raw sequencing data from N=21 healthy Beijing volunteers (NO: 10M/11F mean age 26 years) were downloaded from the Microbial Genome Database System.
	OB	BMI ≥ 30	21				
[107]	NO	BMI 18.5-25	131	V4–V5	Davis, California	DESeq2; Wald Test	
	OW	BMI 25-30	124				

	OB	BMI 30-45	88				Healthy adults (N = 358) were recruited using an 18-bin sampling scheme stratified by age (18–33, 34–49, or 50–65 years), sex, and BMI.
[98]	MHNO	BMI 18.5-25	76	V4	Nashville, Tennessee	Student's t-test	White (N=81), Asian (N=19), Black (N=37), Other (N=13). Excluded: pregnant/lactating, smoking, medications/supplements, clinical diagnoses.
	MHOW	BMI 25-30	34				
	MHOB	BMI ≥ 30	25				
[119]	NO	BMI 18.5-24	30	V3-V4	Beijing, China	Wilcoxon Rank-Sum	Excluded: pregnant/lactating, GI or infectious diseases; cancer; autoimmune disorders; renal dysfunction; weight loss treatment; antibiotic/probiotic use (1 year).
	OB	BMI ≥ 28	30				
[103]	MHNO	BMI 18.5-25	37	V3-V4	Emilia Romagna region and surroundings (Italy)	Wilcoxon Rank-Sum	Premenopausal women. Excluded: T2DM; endocrine, metabolic, mental disorders; renal, hepatic, CNS diseases; cancer; medication, alcohol use; pregnant/lactating; dietary regimen
	MHOB	BMI 25-40	63				
[72]	NO	BMI 18.5-25	24	V2-4-8; V3-6,7-9	Waiʻanae and Pālolo, Oʻahu, Hawaiʻi	Kruskal-Wallis & Wilcoxon Rank-Sum; Spearman Correlation	N=138 participants (aged 16 to 79 years); residents of NHPI-enriched communities.
	OW	BMI 25-30	29				
	OB	BMI ≥ 30	85				
[68]	MHUW	BMI < 18.5		V3-V4	Harbin, China	Welch's t-test; Wilcoxon Rank-Sum	Vegetarians (N=46). Omnivores (N=75). Aged 25-45 years. Exclusion: T2DM; infectious, GI diseases; cancer; antibiotics; pregnant/lactating; smoking; drinking.
	MHNO	BMI 18.5-24					
	MHOW	BMI 24-28					
	MHOB	BMI ≥ 28					
[73]	MHNO	BMI < 25	75	V3-V4-V6	Siracusa, Sicily	Linear and logistic regression (adjusted for	163 industrial workers (aged 47.5 ± 11.4 years, 73% male). Excluded: pregnancy, transplantation,
	OB	BMI ≥ 25	88				

						age, sex, CVD, thyroid diseases)	chronic/end-stage disease, and inflammatory bowel disease.
[90]	MHNO	BMI 19.5-25	10	V3-V4	Guadalajara, Jalisco, Mexico	PERMANOVA; Spearman Correlation	Mexican adults (20F/10M, aged 20-50 years). Excluded: GI/diabetic/metabolic disorders; antibiotics/probiotics/antifungals (3 months)
	MHOW	BMI 25-30	10				
	MHOB	BMI 30-35	10				
[72]	MHNO	BMI < 30	216	V4	Los Angeles, California	DESeq2 (adjusted for race and regional diet)	198F/98M. White (N=110). Hispanic (N=92). Asian (N=65). Black (N=24). AIAN (N=6). OB median age 32 years. NO median age 26 years. Excluded: pregnancy/lactation, extreme exercise, major diagnoses, antibiotic/analgesic use, surgery, hypertension, DM, MetS, tobacco/drug use.
	MHOB	BMI ≥ 30	81				

hvr=Hypervariable region (16S rDNA target). MH=Metabolically healthy. MU=Metabolically unhealthy. UW=Underweight. NO=Non-obese. OW=Overweight. OB=Obese. AIAN=American Indian or Alaska Native. F=Female. M=Male. MetS=Metabolic syndrome. T2DM=Type 2 diabetes. DM=diabetes. NASH=Nonalcoholic steatohepatitis. GI=gastrointestinal. LEfSe=Linear discriminant analysis Effect Size. PERMANOVA=Permutational analysis of variance. STAMP=statistical analysis of taxonomic and functional profiles[143]. Cells are intentionally left blank if the information is unclear.

Filters applied: in the last 10 years.

Search Query: (("Native Hawaiian" OR "Hawaiian" OR "Polynesian" OR "Pacific Islander" OR "Maori" OR "New Zealander" OR "NHPI" OR "Melanesian" OR "Micronesian" OR "Tongan" OR "Samoan" OR "Fijian" OR "Guamanian" OR "Chamorro" OR "Palauan" OR "Niuean" OR "Tokelauan" OR "Cook Islander" OR "Marshallese" OR "Kiribati" OR "Tuvaluan" OR "Nauruan" OR "Papua New Guinean" OR "Rotuman" OR "Pitcairn Islander" OR "Solomon Islander" OR "West Papuan" OR "Moluccan" OR "Kanak" OR "Ni-Vanuatu" OR "Carolinians" OR "Chuukese" OR "I-Kiribati" OR "Kosraean" OR "Pohnpeian" OR "Yapese" OR "Rapa Nui" OR "Tahitian" OR "Wallisian" OR "Futunan")) AND (("gut" OR "fecal" OR "stool" OR "faecal" OR "intestinal")) AND (("microflora" OR "microbiota" OR "microbiome" OR "bacteria" OR "metataxonomic" OR "metagenomic" OR "16S" OR "dysbiosis")) AND (("sequencing" OR "profiling" OR "taxonomics" OR "population" OR "relative abundance" OR "prevalence" OR "community composition" OR "diversity" OR "metagenomic" OR "metataxonomic")) NOT (Review[Publication Type] OR "Meta-Analysis"[Publication Type])

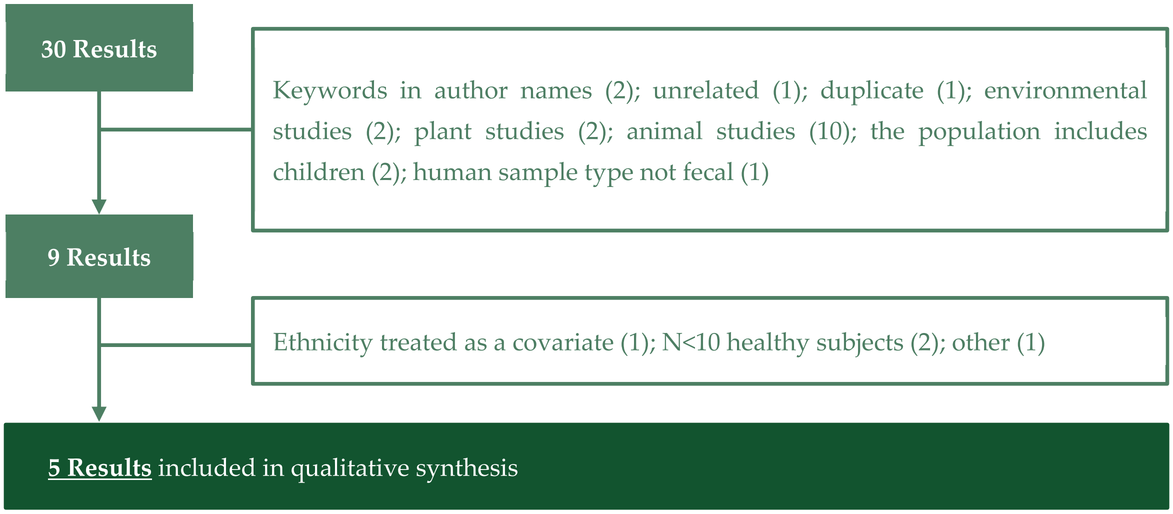

Figure S2. Flowchart of inclusion/exclusion for the (non-comprehensive) review of current literature using the PubMed database (**Table 4**). Place names were not used as search terms to minimize the return of irrelevant results. The publication excluded for “other” reasons was accompanied by an expression of concern.

Table S2. Methodological summary for recent studies on obesity-related trends in 16S-based metataxonomics (2019-2024).

	Summary of study methodology			Summary of study population		
**Ref**	**hvr**	**Location**	**Analysis**	**Race-Ethnicity**	**N**	**Description**
[125]	V1–V3	Honolulu, Hawaii; Los Angeles, California	Beta-binomial regression (adjusted for sex and total fat mass) stratified by self-reported ethnicity	Japanese American	400	1,861 healthy multiethnic cohort (MEC) men and women aged 60–77 years. Excluded: smoking; severe alcohol consumption; amputation; implants; claustrophobia; insulin, thyroid medication; dialysis; severe health conditions; chemo, radiation (6 months); antibiotic use; recent weight change >20lbs; colonoscopy, vaccination (1 month).
				African American	257	
				White	316	
				Latino	325	
				Native Hawaiian	246	
[127]	V2-4-8; V3-6,7-9	O‘ahu, Hawaii	Linear regression	NHPI	47	NHPI-enriched cohort, aged 17-79 years. Predominantly female (62%).
				non-NHPI	21	
[72]	V2-4-8; V3-6,7-9	O‘ahu, Hawaii	Spearman Rank-Order Correlation	NHPI-enriched cohort	138	Stratified by age group: Early Adulthood (16-20 years, N=37), Young Adulthood (21-35 years, N=41), Mid-Adulthood (36-55 years, N=36), and Late Adulthood (56-80 years, N=24). BMI and A1c levels differed between age groups.
[126]	V4	United States	SparseMCMM_HD (controlled for 23 covariates)	API	130	AGP data accessed for adult USA residents. Excluded: BMI>80; height >210cm, <80cm; weight >200kg, <35kg; IBD; DM; pregnant; alcohol, tobacco use; antibiotic use (1 year)
				non-Hispanic Caucasian	2263	
[128]	(WGS)	Waiben and Mer, Torres Strait Islands	ANCOVA (adjusted for age); ACME (adjusted for age, location)	Waiben cohort	50	Participants were drawn equally from Waiben (commercial, administrative center), and Mer (smaller, more remote).
				Mer cohort	50	

API=Asian and Pacific Islanders. AGP=American Gut Project. NAFLD=Non-alcoholic fatty liver disease (liver fat >5.5%). IBD=inflammatory bowel disease. DM=diabetes. WGS=whole genome shotgun metagenomic sequencing. ACME= Average causal mediation effect.
